# HTLV-1 p12 modulates the levels of prion protein (PrP^C^) in CD4^+^ T cells

**DOI:** 10.3389/fmicb.2023.1175679

**Published:** 2023-08-10

**Authors:** Isabela Silva De Castro, Alessandra Granato, Rafael Meyer Mariante, Marco Antonio Lima, Ana Claudia Celestino Leite, Otávio de Melo Espindola, Cynthia A. Pise-Masison, Genoveffa Franchini, Rafael Linden, Juliana Echevarria-Lima

**Affiliations:** ^1^Laboratório de Imunologia Básica e Aplicada, Instituto de Microbiologia Paulo de Góes, Universidade Federal do Rio de Janeiro, Rio de Janeiro, Brazil; ^2^Animal Models and Retroviral Vaccines Section, Vaccine Branch, National Cancer Institute, Bethesda, MD, United States; ^3^Program in Genetics and Genome Biology, Hospital for Sick Children, Toronto, ON, Canada; ^4^Laboratório de Neurogenesis, Instituto de Biofísica Carlos Chagas Filho, Universidade Federal do Rio de Janeiro, Rio de Janeiro, Brazil; ^5^Laboratório de Biologia Estrutural, Instituto Oswaldo Cruz, Fiocruz, Rio de Janeiro, Brazil; ^6^Instituto Nacional de Infectologia Evandro Chagas (INI), Fundação Oswaldo Cruz (Fiocruz), Rio de Janeiro, Brazil

**Keywords:** HTLV-1, PrP^C^, lymphocytes, HTLV-1-infected cells, IFNγ, p12

## Abstract

**Introduction:**

Infection with human T cell lymphotropic virus type 1 (HTLV-1) is endemic in Brazil and is linked with pro-inflammatory conditions including HTLV-1-associated myelopathy/tropical spastic paraparesis (HAM/TSP), a chronic neuroinflammatory incapacitating disease that culminates in loss of motor functions. The mechanisms underlying the onset and progression of HAM/TSP are incompletely understood. Previous studies have demonstrated that inflammation and infectious agents can affect the expression of cellular prion protein (PrP^C^) in immune cells.

**Methods:**

Here, we investigated whether HTLV-1 infection affected PrP^C^ content in cell lines and primary CD4^+^cells *in vitro* using flow cytometry and western blot assays.

**Results:**

We found that HTLV-1 infection decreased the expression levels of PrP^C^ and HTLV-1 *Orf I* encoded p12, an endoplasmic reticulum resident protein also known to affect post-transcriptionally cellular proteins such as MHC-class I and the IL-2 receptor. In addition, we observed a reduced percentage of CD4^+^ T cells from infected individuals expressing PrP^C^, which was reflected by IFN type II but not IL-17 expression.

**Discussion:**

These results suggested that PrP^C^ downregulation, linked to both HTLV-1 p12 and IFN-γ expression in CD4^+^ cells, may play a role in the neuropathogenesis of HTLV-1 infection.

## 1. Introduction

Human T cell lymphotropic virus type 1 (HTLV-1) is the etiological agent of the adult T cell leukemia/lymphoma (ATLL) malignancy (Poiesz et al., 1980; Gallo et al., 1986; Uchiyama, 1996; Uchiyama, 1997) and HTLV-1-associated myelopathy/tropical spastic paraparesis (HAM/TSP) (Gessain et al., 1985; Osame et al., 1986), a chronic, progressively disabling disease characterized by demyelination, axonal loss, neuronal degeneration, and gliosis. The main site of neurodegeneration is the thoracic spinal cord, leading to a slowly progressive spastic paraparesis including low back pain and bowel, urinary, and urinary and sexual dysfunction (Leite et al., 2004; Araujo and Silva, 2006). High prevalence areas of HTLV-1 infection include Latin America, Southern Japan, and Central and Western Africa (Gonçalves et al., 2010). In Brazil, the prevalence of HTLV-1 infection among blood donors is approximately 0.45% among blood donors, and 0.32% among pregnant women, which may correspond to approximately 1 million infected people (Araújo Ade and de Andrada-Serpa, 1996; Proietti et al., 2005; Vieira et al., 2021).

The HTLV-1 viral genome encodes structural genes (Gag, Pol, and Env) and five open reading frames (orfs) collectively referred to as the pX region. Tax and Rex viral proteins are encoded by *Orfs IV* and *III*, respectively. The Tax protein has been identified as a potent activator of a variety of transcription pathways and has been related to T cell transformation (Currer et al., 2012; Mohanty et al., 2020). The Rex protein is associated with the post-transcriptional regulator of viral expression (Hidaka et al., 1988; Inoue et al., 1991). *Orf I* and *Orf II* encode genes whose functions are primarily associated with the modulation of host immune responses (Edwards et al., 2011; Moles et al., 2019; Sarkis et al., 2019; Omsland et al., 2020). *Orf I* encodes the endoplasmic reticulum (ER) resident p12 precursor protein, which can be further processed into p8 (Berneman et al., 1992; Koralnik et al., 1992, 1993; Fukumoto et al., 2007), whereas *Orf II* encodes the p13 and p30 proteins (Ciminale et al., 1992; Koralnik et al., 1992; Ciminale et al., 1999; Bartoe et al., 2000; Nicot et al., 2004).

The p12 protein is localized in the ER and Golgi complex (Koralnik et al., 1992), and it is required to efficiently infect primary human T lymphocytes (Albrecht et al., 2000) and dendritic cells *in vitro* and macaques *in vivo* (Valeri et al., 2010). Moreover, p12 promotes MHC class I proteasome degradation, reducing HTLV-1 antigen presentation, and recognition of HTLV-1-infected cells by cytotoxic CD8^+^ T cells (Johnson et al., 2001; Pise-Masison et al., 2014). HTLV-1 infection induces the activation of T lymphocytes, leading to spontaneous proliferation, expression of molecules with associated cellular activation, and production of pro-inflammatory cytokines (Prince et al., 1994; Ishikawa et al., 2013; Novaes et al., 2013; Coutinho et al., 2014). This phenomenon can be related to the transactivation of genes by Tax and by the ability of p12 to induce activation of the NFAT pathway (Albrecht et al., 2002) and the JAK/STAT5 pathway, thereby reducing the cell requirement of IL-2 (Nicot et al., 2001).

While p12 increases T cell activation (Albrecht et al., 2002; Ding et al., 2002, 2003; Kim et al., 2003, 2006), its cleavage product p8(I) is recruited to the immunologic synapse following TCR engagements, suggesting downregulation of TCR signaling (Fukumoto et al., 2009; Prooyen et al., 2010). The p8 protein gains access to neighboring cells via cellular conduit and favors cell-to-cell transmission by inducing T cell clustering through LFA-1 expression and conduit formation (Prooyen et al., 2010; Donhauser et al., 2020). The expression of p8 is also required in virus-infected cells for escape from immune recognition by cytotoxic CD8^+^ T cells (Pise-Masison et al., 2014; Gutowska et al., 2022), raising the hypothesis that p8, by entering CD8 cells and downregulating TCR, may affect the strength of the immunological synapse and CD8^+^ T cell killing.

The prion protein (PrP^C^) is a glycoprotein bound to the external surface of cells by a glycosylphosphatidylinositol (GPI) anchor. PrP^C^ is well known in neuropathology since its post-translational conversion can lead to a transmissible spongiform encephalopathy-associated protein isoform (PrP^Sc^) that is characterized by numerous chemical modifications including sialic acid residues attached to glycosyl inositol phospholipid anchor (Stahl et al., 1992). PrP^C^ is highly conserved and found constitutively in cells such as those in the nervous and immune systems, where it is involved in a plethora of biological events, such as oxidative stress, cell survival and death, cell differentiation, cell adhesion, T cell activation, myelin maintenance, and synaptic transmission (Spielhaupter and Schätzl, 2001; Ford et al., 2002; Isaacs et al., 2006; Westergard et al., 2007; Linden, 2017).

PrP^C^ is expressed by many cells of the immune system such as T and B lymphocytes, dendritic cells, NK cells, macrophages, and monocytes (Dürig et al., 2000; Li et al., 2001; Thielen et al., 2001). In T lymphocytes, its expression increases with cell activation. Mabbott et al. (1997) demonstrated that lymphocytes from PrP^C−/−^ mice have a reduced proliferation compared with T lymphocytes of wild-type (PrP^C+/+^) mice when stimulated by Concanavalin A (Mabbott et al., 1997). In humans, CD8^+^ T cells have a significantly higher PrP^C^ expression than CD4^+^ T cells, and IFN-γ significantly upregulates PrP^C^ expression on monocytes in a concentration and time-dependent manner (Dürig et al., 2000).

Using goats naturally born without PrP^C^ demonstrated that PrP^C^, Malachin et al. (2017) deficiency impacts interferon signaling by upregulating type I IFN genes. Moreover, after lipopolysaccharide injection, these animals exhibited longer persistence of clinical symptoms (Salvesen et al., 2017). Thus, the expression of PrP^C^ might play an important role in viral and bacterial infections.

The expression of the PrP^C^ pattern was evaluated in the cerebrospinal fluid (CSF) obtained from HAM/TSP patients, but no significant difference was observed (Torres et al., 2012). However, recently Souza et al. (2021) demonstrated that higher levels of soluble PrP^C^ were found in CSF in addition to higher levels of CCL2 and neopterin. The study of different elements of the immune response and the alterations induced by viral proteins is essential for the comprehension of neurological pathology. Thus, we analyzed whether HTLV-1 infection affects the content and expression level of PrP^C^ in CD4^+^ T cells *in vitro* and *in vivo* in HTLV-1-infected individuals. We found that HTLV-1 infection decreases PrP^C^ levels *in vitro* via the expression of the HTLV-1 p12 protein encoded from the viral *Orf I* gene. In addition, the frequency of CD4^+^ cells expressing PrP^C^ was decreased in CD4^+^ cells of peripheral blood from people living with HTLV-1 compared with non-infected individuals. Reduced PrP^C^ levels did not correlate with CD25^+^ expression or proviral load in CD4^+^ T cells, suggesting other mechanisms may be involved. We also found higher IFN-γ expression in cells with reduced PrP^C^ expression. Our studies suggest that the expression of PrP^C^ is regulated in T lymphocytes upon HTLV-1 infection and may impact susceptibility to the development of HAM/TSP.

## 2. Materials and methods

### 2.1. Subjects

All experimental procedures were performed in accordance with Brazilian Resolution 196/96 and 466/12 of the National Health Council published by the Brazilian National Research Ethics Commission (Comissão Nacional de Ética em Pesquisa/CONEP). The study was approved by the institutional review board (CAAE: 53518416.9.0000.5262). This study involved 27 HTLV-1-non-infected donors, 26 asymptomatic carriers (AC), and 24 HAM/TSP patients from the cohort of the Instituto Nacional de Infectologia Evandro Chagas (INI) of the Fundação Oswaldo (Fiocruz), Rio de Janeiro, Brazil. Clinical HAM/TSP diagnosis was performed following the World Health Organization guidelines (WHO, 2021). Patients were excluded from the study when diagnosed with co-infection with other viruses (HIV, HBV, HCV, and HTLV-2).

### 2.2. PBMC isolation

The PBMCs were obtained from heparinized whole blood by Ficoll-Histopaque 1077 (Sigma–Aldrich, United States) gradient centrifugation (400 g/40 min). Following this, PBMCs were washed two times with PBS and resuspended with Roswell Park Memorial Institute medium-1640 (RPMI-1640) (LGC Biotechnologia, Brasil, or Gibco/Thermo Fisher Scientific, United States) and supplemented with FBS 10% (FBS; Gibco/Thermo Fisher Scientific, United States), penicillin (1,000 UI/mL), and streptomycin (100 mg/mL), both from LGC Biotechnologia, Brazil, or Gibco/Thermo Fisher Scientific, United States.

### 2.3. Cell lines and co-culture

All cells were cultured in RMPI-1640 supplemented with 10% FBS, penicillin (100 UI/ml), and streptomycin (100 mg/ml). Cells were cultivated at 37°C in a humidified atmosphere with 5% CO_2_ and were passaged twice a week. HTLV-1 transformed cell lines MT-2 and C91PL were provided by INI, FIOCRUZ, RJ, Brazil. The Jurkat cell line was generously provided by Dra. Ana Lucia Giannini of UFRJ. The HTLV-1-infected T cell lines TL-Om-1 and ED40515(−) (from ATL patients) and 6-thioguanine-resistant human lymphoblastoid B cell line (729-6) were described previously (Maeda et al., 1985; Albrecht et al., 2000; Johnson et al., 2001; Edwards et al., 2011).

MT-2 cells were irradiated at 2000 rad, using the Gammacell^®^ 220 Excell equipment. In parallel, 1×10^6^ target cells (Jurkat) were stained with 1 μM CFSE (Life Technologies/Thermo Fisher Scientific) for 15 min at 37°C. After that, cells were washed two times with PBS and centrifuged at 200 g for 7 min. Irradiated MT-2 cells were co-cultured with Jurkat cells (5×10^5^; 1:1). After 48 h or 96 h at 37°C in a humidified atmosphere with 5% CO_2_, CFSE-positive cells were sorted using Moflo cell sorter (Dako Cytomation/Beckman Coulter). Confirmation of infection was performed through PCR of the viral Tax gene. In addition, cells were labeled as described below, and the cells were analyzed by flow cytometry.

### 2.4. Transfection and silencing

Jurkat cells (3 × 10^6^) were transfected with 2 μg of DNA plasmid, using Amaxa^®^ Cell Line Nucleofector^®^ Kit V. The HTLV-1 molecular clones pAB WT, p12(G29S), p8(N26), and p12KO and the *Orf I* expression constructs pME-G29S and pME-WT were described previously (Fukumoto et al., 2007; Prooyen et al., 2010; Valeri et al., 2010; Pise-Masison et al., 2014). Electroporation was performed by Nucleofector II Amaxa equipment (Lonza, Switzerland), according to the manufacturer’s instructions.

PrP^C^ expression was silenced in MT-2 cells using human PrP^C^ siRNA (PrP siRNA(h):sc-36318; Santa Cruz Biotechnology). Cells were cultured in Optimem media (Thermo Fisher Scientific) supplemented with 10% FBS for 24 h at 37°C in a humid atmosphere with 5% CO_2_. The siRNA transfection was performed with 60–80% confluence of cultures, using Lipofectamine 2000 (Invitrogen/Thermo Fisher Scientific), according to the manufacturer’s instructions. mRNA PrP^C^ silencing was determined by qRT-PCR (Erdogan et al., 2013).

### 2.5. Flow cytometry

For prion protein, we used monoclonal antibody (Clone SAF 32, 1:50) from Santa Cruz Biotechnology. 5×10^5^ cells were incubated with SAF32 for 30 min at 4°C. Then, cells were washed with PBS + 5% FBS, centrifuging for 7 min at 200 g at 4°C. After that, cells were stained with a secondary anti-mouse IgG antibody conjugated to fluorochromes Alexa Fluor 488 or 647 (Life Technology/Thermo Fisher Scientific) diluted 1:1000 (v/v). After 30 min at 4°C, cells were washed with PBS + 5% FBS, centrifuging for 7 min at 200 g at 4°C. Cell phenotyping was performed after PrP^C^ was stained using the flow cytometry technique, using monoclonal antibodies from ExBio anti-human CD3 conjugated to APC or FITC (1:100), anti-human CD4 conjugated to APC (1:100), anti-human CD25 conjugated to PE (1:50); from Biolegend, human CD3 conjugated to BV510 (1:100), anti-human CD4 conjugated to BV785, anti-human CD8 conjugated to PerCP (1:100), anti-human IFN-γ conjugated to Alexa Fluor 488 (1:50), anti-human IL-17 conjugated to PE (1:50), anti-human IL-17 conjugated to PE (1:50), anti-human TNF-α conjugated to BV600 (1:50), anti-human IL-6 conjugated to PECy7 (1:50), anti-human IL-4 conjugated to APC (1:50), and anti-human IL-10 conjugated to BV421 (1:50). 5×10^5^ cells were incubated with antibodies for 30 min at 4°C. Then, cells were washed with PBS + 5% FBS, centrifuging for 7 min at 200 g at 4°C. For IL-17 and IFN-γ labeling, PBMCs (5×10^5^/well) were stimulated *in vitro* in the presence or absence of Ionomycin (250 ng/ml; Sigma–Aldrich/Merck) and PMA (20 ng/ml; Sigma–Aldrich/Merck) for 14 h at 37°C in a humidified atmosphere in 5% CO_2_, with Monensin (5 μM; Sigma–Aldrich/Merck) at least 4 h. After that, cells were washed with PBS and labeled with different surface antibodies as described above. Then, the cells were fixed with 2% paraformaldehyde, washed with PBS, and permeabilized with a permeabilization solution (eBioscience/Thermo Fisher Scientific) for 30 min at room temperature. After that, the cells were labeled with the antibodies for 30 min at room temperature. Following this, cells were washed, and 30,000 events were acquired for analysis of the percentage and mean fluorescence intensity, using FACSCalibur or CANTO (BD) cytometers. For analysis, Summit V4.3 and FlowJo V10 software were used.

### 2.6. p19 quantification by ELISA

The production of HTLV-1 in the supernatants of the HTLV-1-infected cell cultures was assessed by measuring the amount of p19 Gag protein by enzyme-linked immunosorbent assay, according to the manufacturer’s instructions (Zeptometrix, Buffalo, NY, United States). The supernatant of cell cultures was centrifuged, and the cell pellet was discarded. Supernatants were treated with lysis buffer, mixed well, and added to pre-coated plates. The plate was incubated for 2 h at 37°C and washed six times using a washing buffer. HTLV-1 detector antibody was added and incubated for 1 h at 37°C. The plates were washed as previously described. Peroxidase working solution was added and incubated for 1 h at 37°C; plates were washed again as previously described. The substrate was added for 30 min at room temperature. A stop solution was added. Plates were read at 450 nm in a microplate reader.

### 2.7. Immunofluorescence microscopy

2×10^5^ cells were centrifuged at 800 rpm for 3 min on glass slides (Thermo Fisher Scientific Scientific, Waltham, MA, United States). Cytospins were fixed with 2% paraformaldehyde for 1 h at room temperature. Next, unspecific binding was blocked by incubation with blocking buffer (PBS + FBS 5%) for 2 h. Glass slides were washed three times and incubated overnight with SAF32 mAb diluted 1:50 (v/v). Following this, cytospins were washed three times and incubated with secondary antibody anti-mouse IgG conjugated to Alexa Fluor 647 (Invitrogen/Thermo Fisher Scientific) diluted 1:1000 (v/v) for 1 h at room temperature. Cytospins were washed three times and incubated with DAPI (2 mg/ml; Sigma–Aldrich). After 5 min at room temperature, glass slides were washed, and fluorescence microscopy was performed using a confocal microscope (Leica TCS SP5 com AOBS) with 63X objective.

### 2.8. Western blot assay

Cells were incubated in lysis buffer (Tris–HCl [50 mM, pH 7.4], Sodium deoxycholate [25%], EDTA [1 mM], Aprotinine [10 μg/mL], Leupeptin [10 μg/mL], Pepstatin [10 μg/mL] from Sigma–Aldrich/Merck, PMSF [1 mM] from Thermo Fisher Scientific, NaCl [150 mM] from Vetec, and Nonidet P-40 [1%] from Abcam) for 15 min and centrifuged at 10,000 g for 20 min at 4°C. The supernatant was harvested, and the total protein concentration was measured by the Lowry method, using albumin (Sigma–Aldrich/Merck) as standard. The protein extracts were separated by SDS-PAGE (NuPAGE^™^ 4–12% Bis-Tris Protein Gels, 1.0 mm, Thermo Fisher Scientific) for 1 h at 100A, approximately, and then transferred to a 7 cm × 8.4 cm, 0.45 μm pore size, hydrophobic PVDF (Immobilon-P PVDF, Millipore Sigma Millipore), previously activated with methanol for 10 s. After 1 h at 140 mA, the membranes were blocked with 5% milk in TBT-T for 1 h at room temperature. The membranes were incubated overnight at 4°C with primary antibodies SAF32 (1:500) or Anti-HA (1:1000) in PBS containing 0.1% Tween-20 (BioRad) and 0.25% milk. Membranes were washed in PBS + 0.1% Tween-20 and exposed to a secondary antibody anti-Mouse HRP (1:10,000, Sigma–Aldrich/Merck) for 1 h at room temperature. Protein levels were detected with SuperSignal West Pico Substrate or SuperSignal West Femto Substrate (Thermo Scientific Pierce), according to the manufacturer’s instructions. The membranes were revealed in a dark chamber in X-ray film (CL-XPosure Film-Thermo Scientific) in a reveling cassette for 3 to 5 min. The PDVF membranes were stripped and relabeled with anti-β-tubulin (1:1000, Sigma–Aldrich/Merck) for loading control. Bands were quantified using ImageJ software.

### 2.9. Real-time PCR (qRT-PCR)

RNA extraction, cDNA synthesis, and qRT-PCR were performed according to Pinheiro et al. (2015). In brief, RNA was extracted using TRIzol^®^ reagent (Invitrogen/Thermo Fisher Scientific), according to the manufacturer’s instructions. Following this, the cDNA was synthesized using the cDNA first strand synthesis kit (Fermentas), according to the manufacturer’s instructions. The qPCR was performed using the primers for the human PrPc gene (*prnp*), forward 5′-ACCCACAGTCAGTGGAA-3′ and reverse 5′- TATGATGGGCCTGCTCA-3′ and *gapdh*, forward 5′-CCAGATCATGTTTGAGACC-3′ and reverse 5′- ATGTCACGCACGATTTCCC-3′ (Invitrogen/Thermo Fisher Scientific). The quantification of *prnp* expression was performed using SYBR Green Real-Time PCR Master Mix (Applied Biosystem/Thermo Fisher Scientific). The cDNA amplifications were performed in an ABI 7500 system (Applied Biosystems), under thermocycling conditions for the reaction followed by 50°C for 2 min, 95°C for 10 min, and 40 cycles of 95°C for 15 s and 60°C for 1 min. The expression level of each gene was normalized to the expression level of the endogenous control (*gapdh*).

### 2.10. Proviral load and detection of HTLV-1-infected cells

DNA was extracted from PBMC with the QIAamp DNA blood mini kit (Qiagen), and DNA was eluted in 30 μl. HTLV-1 proviral load was determined by quantitative PCR in a Rotor-Gene Q instrument (Qiagen), using the Rotor-Gene Probe PCR kit (Qiagen), according to the manufacturer’s instructions. Primers and 5′-FAM and 3′-TAMRA-labeled TaqMan^®^ probes (Sigma–Aldrich) for the HTLV-1 *tax* and the human *β-globin* genes, as previously described (Silva et al., 2007), were used in independent reactions with 5 μl of DNA. HTLV-1 proviral load was calculated as *tax* copies/(*β-globin* copies/2), and the results are shown as infected monocytes per 100,000 cells. Qualitative PCR was performed with 10 μl of DNA in 50 μl reactions using the HotStar Taq Plus PCR kit (Qiagen), following the manufacturer’s instructions, using the same primers for HTLV-1 *tax* and human *β-globin* genes. The amplification cycle consisted of enzyme activation at 95°C for 5 min, 45 cycles of denaturation at 95°C for 30s, annealing at 60°C for 30s, extension at 72°C for 30s, and a final extension step at 72°C for 10 min. PCR products were electrophoresed in 2% agarose gel stained with GelRed^®^ (Biotium) in 1× Tris-Borate-EDTA buffer (Invitrogen) at 100 V for 90 min. Amplification of HTLV-1 *tax* results in a 159 bp PCR product.

### 2.11. Statistical analysis

The one-dimensional probability distributions of samples were analyzed by Kolmogorov–Smirnov test. After that, statistical analysis was performed by one-way analysis of variance followed by Bonferroni’s post-test for samples with normal distribution, or Kruskal–Wallis analysis followed by Dunn’s post-test. Statistical analysis was performed by unpaired *t*-test with Welch’s correction or Mann–Whitney *U* test to two groups of samples with normal or without normal distribution, respectively. Correlations were analyzed by Spearman’s or Pearson’s rank correlation coefficient. The statistical analysis was performed using GraphPad Prism 8 software and values of *p* < 0.05 were considered statistically significant.

## 3. Results

### 3.1. Reduced PrP^C^ content in HTLV-1-infected cell lines

**Figure 1:**
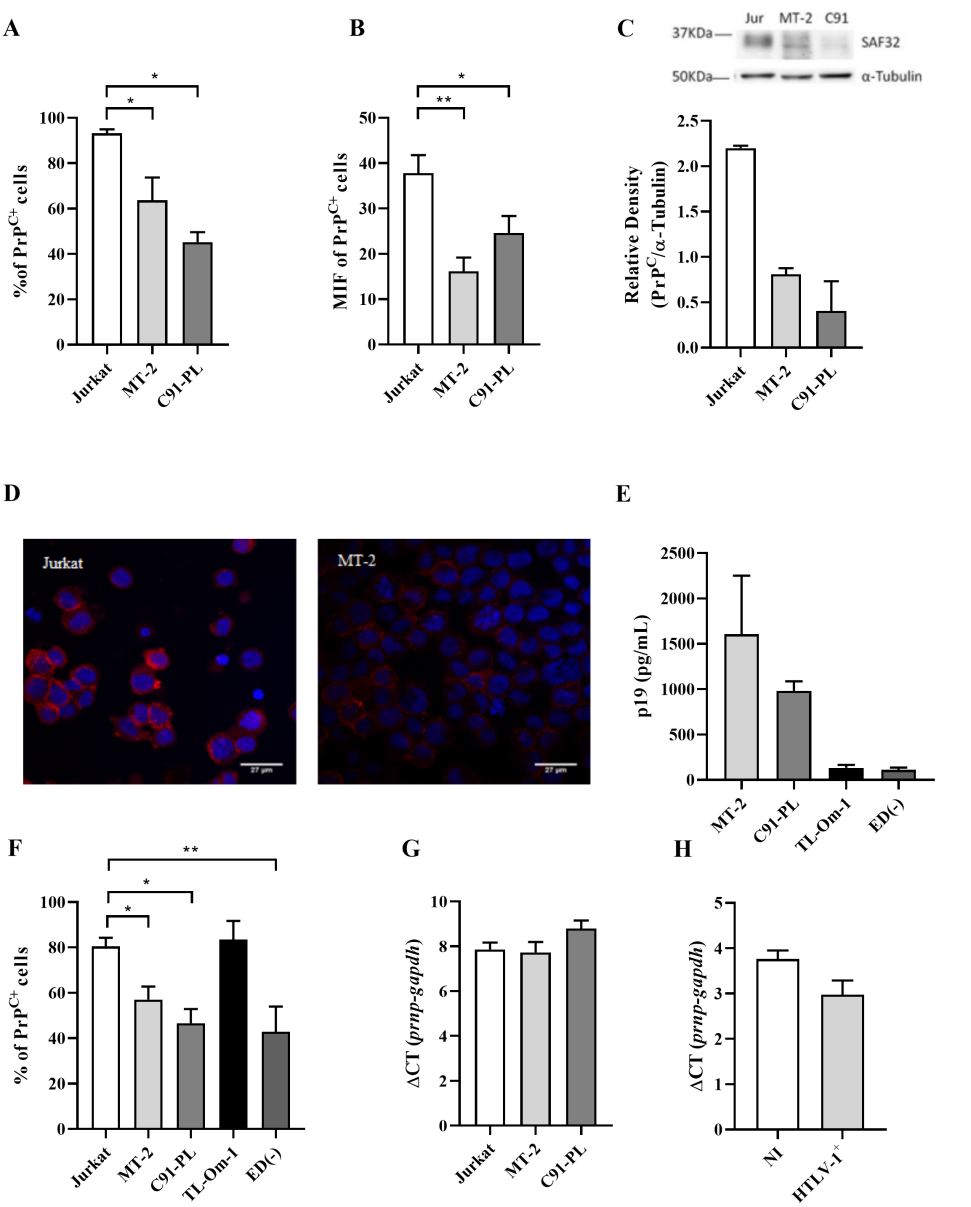
PrP^C^ levels in cell lines. 5×10^5^ cells of different cell lines were stained with the antibody SAF32 as described in the methods section. **(A,B)** PrP^C^ levels were analyzed by flow cytometry in Jurkat cells (non-infected cells), MT-2 cells, and C91-PL cells (HTLV-1-infected cells) to compare the percentage of PrP^C+^ and the mean fluorescence intensity (MFI) for PrP^C^ labeling in different cells. Statistical analysis was performed using the ANOVA statistical test following Bonferroni’s post-test. The means were considered significantly different when *p < 0.05, **p < 0.002, and ***p < 0.0001. **(C)** 30 μg of total proteins from 10^6^ Jurkat, MT-2, and C91-PL cells were separated in SDS-PAGE and transferred to nitrocellulose membrane to analyze PrP^C^ content by Western blot, using α-tubulin as a constitutive protein as described above. **(D)** 2×10^5^ Jurkat and MT-2 cells were centrifuged in a cytospin, fixed with 2% paraformaldehyde and blocked with PBS + 5% FBS. Cells were incubated overnight with SAF32 antibody and then incubated with anti-mice IgG secondary antibody conjugated with Alexa Flour 647 as described above. The nuclear stain was performed with DAPI (2 mg/ml). Microscopy was performed using a fluorescence microscope with a 63× objective. **(E)** 5×10^5^ cells/mL were incubated for 24 h for the supernatant collection and analysis of the p19 concentration determined by ELISA, according to the manufacturer’s instructions. **(F)** PrP^C^ expression was analyzed by flow cytometry in Jurkat cells (non-infected cells), MT-2 cells, C91-PL cells, and HTLV-1-infected cells without viral particle release (p19^neg/low^) as TL-Om-1 and ED40515(−) to compare the percentage of PrP^C+^. Statistical analysis was performed using the ANOVA statistical test following Bonferroni’s post-test. The means were considered significantly different when *p < 0.05 and **p < 0.002. **(G)** Total RNA was extracted from 10^6^ cell lines (Jurkat, MT-2, and C91-PL) or **(H)** sorted CD4^+^ T lymphocytes from PBMCs of non-infected cells and HTLV-1 carriers (HTLV-1^+^). RNA was extracted and then subjected to an RT-PCR reaction with random primers to obtain cDNA. With the cDNA, qPCR reactions were performed with specific primers for the *gapdh* mRNA (housekeeping gene) and *prnp* (PrP^C^ gene).

To assess whether the PrP^C^ protein levels are affected by HTLV-1 infection, we evaluated by flow cytometry PrP^C^ content in the well-established T lymphoid HTLV-1-infected cell lines, MT-2 and C91-PL, compared with the HLTV-1 negative immortalized T cell line Jurkat. While 93.23% of Jurkat cells were PrP^C^-positive (PrP^C+^), the HTLV-1-infected cell lines had a significantly reduced percentage of PrP^C+^ cells; approximately 63.72% and 45.25% of MT-2 and C91-PL, respectively (Figure 1A and Supplementary Figure S1). Moreover, the HTLV-1-infected cells exhibited a lower mean fluorescence intensity, indicating a reduced level of PrP^C^ per cell (Figure 1B). These results were confirmed by Western blot and fluorescence microscopy. Evaluation of HTLV-1-infected cell lysates corroborated the results found by flow cytometry (Figure 1C), as well as fluorescence microscopy assays where MT-2 cells presented reduced levels of PrP^C^ compared to Jurkat cells (Figure 1D). TL-Om-1 and ED40515(−) cells are HTLV-1-infected T cell lines established from ATL patients (Maeda et al., 1985; Forlani et al., 2021). However, these cells do not express Tax or release virus as shown by low levels of p19 release into the supernatant (Figure 1E). Similar to MT-2 and C91-PL, ED40515(−) presented reduced PrP^C^ levels compared to Jurkat, but TL-Om-1 cells expressed levels similar to Jurkat (Figure 1F). These results suggest that Tax expression may not be linked to PrP^C^ content.

To determine if reduced PrP^C^ protein levels were related to reduced mRNA expression, we used RT-PCR to analyze PrP^C^ gene expression (*prnp*). Interestingly, the downregulation of PrP^C^ content did not depend on the inhibition of *prnp* gene transcription. The levels of PrP^C^ mRNA were similar in infected and uninfected cells (Figures 1G,H), suggesting that the PrP^C^ expression was regulated post-transcriptionally.

### 3.2. Altered percentage of CD4^+^ PrP^C+^ cells of HAM/TSP and AC patients

**Figure 2:**
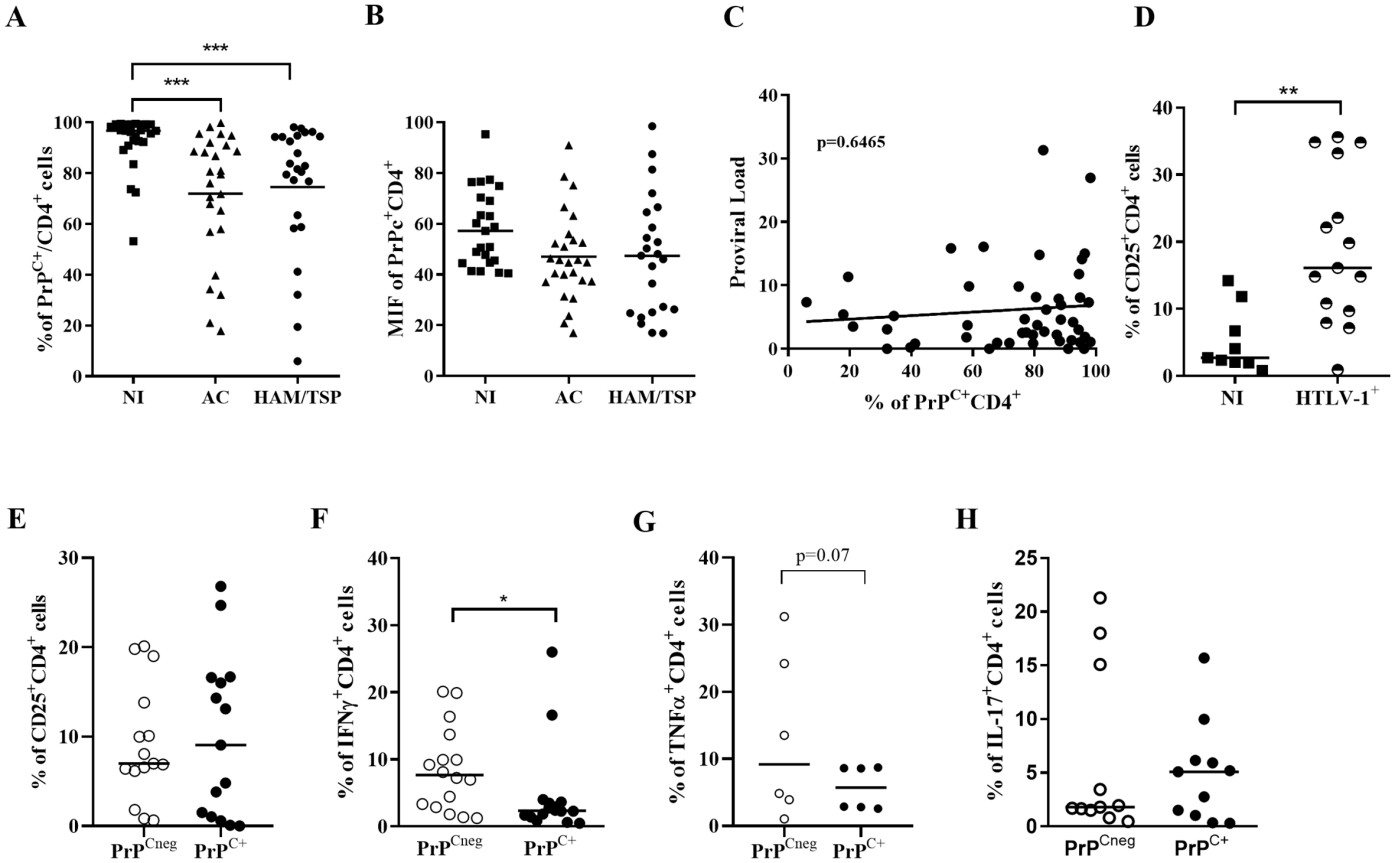
Percentage of CD4^+^PrP^C+^ cells from people living with HTLV-1. **(A,B)** 5×10^5^ cells of PBMCs obtained from non-infected individuals (NI, *n* = 27), asymptomatic carriers (AC, *n* = 26), and HAM/TSP (*n* = 24) patients were stained with the antibody SAF32 and lineage-specific antibodies as described in the methods section. PrP^C^ levels were analyzed by flow cytometry in CD4^+^ cells from three groups to compare the percentage of PrP^C+^ and the mean fluorescence intensity for PrP^C^ labeling in different groups. ***p=0.0008 determined by Kruskal-Wallis test. **(C)** Spearman’s correlation test between the percentage of PrP^C^ in CD4^+^ cells and proviral load, *r* = 06792 and *p* = 06465. **(D)** Percentage of CD25^+^ in CD4^+^ cells from PBMCs of people living with HTLV-1 (*n* = 15) and non-infected donors, *n* = 9. **p = 0.0013 determined by Mann–Whitney test. **(E)** Percentage of CD25^+^ in PrP^Cneg^ CD4^+^ cells or PrP^C+^ CD4^+^ cells from PBMCs of people living with HTLV-1, *n* = 15. **(F)** Percentage of IFN-γ^+^ in PrP^Cneg^ CD4^+^ cells or PrP^C+^ CD4^+^ cells from PBMCs of people living with HTLV-1, *n* = 16. *p = 0.0228 determined by Mann–Whitney test. **(G)** Percentage of TNF-α^+^ in PrP^Cneg^ CD4^+^ cells or PrPC^+^ CD4^+^ cells from PBMCs of people living with HTLV-1, *n*=6. (p=0.07 determined by Mann-Whitney test). **(H)** Percentage of IL-17^+^ in PrP^Cneg^ CD4^+^ cells or PrP^C+^ CD4^+^ cells from PBMCs of people living with HTLV-1, *n* = 11. Each symbol represents one donor, and the bar indicates the median value.

Next, we evaluated if PrP^C^ levels were also downregulated in CD4^+^ T lymphocytes obtained from the peripheral blood of people living with HTLV-1. We observed by flow cytometry that CD4^+^ T lymphocytes from infected individuals, asymptomatic carriers (AC) or those with HAM/TSP, display a significant reduction in the percentage of cells that express PrP^C^ compared with cells from non-infected individuals (Figure 2A). We observed that approximately 72% of CD4^+^ T cells of AC donors and 74% of CD4^+^ T cells of HAM/TSP patients expressed PrP^C^, while 92% of CD4^+^ T cells of non-infected individuals expressed PrP^C^. Similarly, HAM/TSP and AC patients presented similar percentages of CD4^+^PrP^C+^ lymphocytes. However, we did not observe any reduction in PrP^C^ amount per cell, suggesting that the PrP^C+^ CD4^+^ T lymphocytes from people living with HTLV-1 expressed equivalent levels of this protein (Figure 2B). Moreover, a correlation analysis between the percentage of PrP^C+^ (SAF32) and the proviral load was performed, but no correlation was found (Figure 2C). The results suggest that HTLV-1 infection alters PrP^C^ levels in stably infected cell lines and peripheral blood CD4^+^ T lymphocytes from infected individuals. In CD4^+^ T lymphocytes, the downregulation of PrP^C^ also did not depend on the inhibition of *prnp* gene transcription (Figure 1H), reinforcing that the PrP^C^ was regulated post-transcriptionally.

As described by Prince et al. (1994), HTLV-1 infection induces the activation of CD4^+^ T lymphocytes and presents a phenomenon of spontaneous proliferation, which is accompanied by increased expression of the α chain of the IL-2 receptor, CD25 (Al-Fahim et al., 1999; Novaes et al., 2013). Therefore, we investigated the percentage of PrP^C+^ in CD4^+^CD25^+^ cells from people living with HTLV-1. Consistent with those results, we detected a higher percentage of CD4^+^CD25^+^ lymphocytes in PBMCs from HTLV-1 carriers compared to controls (Figure 2D). Interestingly, we did not observe differences between the percentage of CD4^+^CD25^+^ of PrP^C^-negative (PrP^Cneg^) and PrP^C^-positive (PrP^C+^) lymphocytes obtained from people living with HTLV-1 (Figure 2E).

In addition, Starling et al. (2015) suggested that a pro-inflammatory profile can be used as a biomarker for HAM/TSP and include IFN-γ. Dodon et al. (2004) detected the presence of mRNA for IL-17 in HTLV-1-infected cell lines. Thus, we evaluated the production of IFN-γ and IL-17 by CD4^+^ PrP^Cneg^ and PrP^C+^. In Figure 2F, we observed a significantly higher percentage of IFN-γ expressing cells in CD4^+^ PrP^Cneg^ (median = 7.7%) than in PrP^C+^ cells (median = 2.3%) in people living with HTLV-1 (Supplementary Figure S2). Moreover, the frequency of TNFα^+^ CD4^+^ PrP^Cneg^ cells was discreetly higher than TNFα^+^ CD4^+^ PrPC^+^ cells (Figure 2G). However, we did not observe significant differences in the percentage of IL-17^+^CD4^+^PrP^Cneg^ and IL-17^+^CD4^+^PrP^C+^, IL-6^+^CD4^+^PrP^Cneg^ and IL-6^+^CD4^+^PrPC^+^, IL-4^+^CD4^+^PrP^Cneg^ and IL-4^+^CD4^+^PrPC^+^, or IL-10^+^CD^+^PrP^Cneg^ and IL-10^+^CD4^+^PrPC^+^ cells from the same group of donors (Figure 2H and Supplementary Figure 3).

### 3.3. HTLV-1 infection modulates PrP^C^

**Figure 3:**
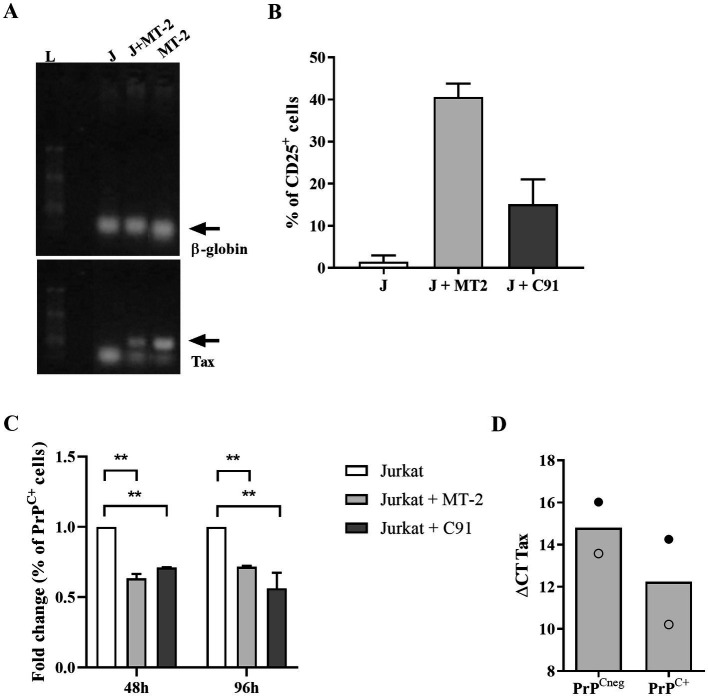
PrP^C^ levels in target cells after HTLV-1 infection. 10^6^ Jurkat cells (target cells) were incubated with 1 μM of CFSE for 15 min at 37°C and washed two times with PBS. Next, CFSE^+^ target cells were incubated with 10^6^ cells of the MT-2 or C91-PL lineage, previously irradiated (2000 rads). **(A)** After 48 h or 96 h, PCR was used to detect a fragment of 159-bp of HTLV-1 tax or β-globin genes in DNA samples obtained from Jurkat cells (J), MT-2 cells (not irradiated) and CFSE^+^ Jurkat cells co-cultivated with MT-2 cells (J + MT-2). L = ladder. **(B)** 5×10^5^ cells of different cell lines were stained with the antibody anti-human CD25 conjugated with PE or **(C)** SAF32 as described in the methods section. PrP^C^ expression was analyzed by flow cytometry in Jurkat cells (non-infected target cells), Jurkat cells co-cultivated with MT-2 cells, and C91-PL cells to compare the fold change in the percentage of PrP^C+^ after HTLV-1 infection. Statistical analysis was performed using the ANOVA statistical test following Bonferroni’s post-test. The means were considered significantly different when **p < 0.0013. **(D)** MT-2 cells were stained with SAF32 antibody as described above to isolate PrP^Cneg^ cells and PrP^C+^ cells by FACS. RNA of PrP^Cneg^ cells and PrP^C+^ cells was extracted and then subjected to an RT-PCR reaction with random primers to obtain cDNA. With the cDNA, qPCR reactions were performed with specific primers for the *gapdh* mRNA (housekeeping gene) and tax gene. The means of two independent experiments are represented in the graph and each symbol (○, ●) corresponds to one experiment.

To confirm the effect of HTLV-1 infection on PrP^C^ content, Jurkat cells were infected by co-culture with MT-2 or C91-PL cells, as described in the methodology section. As shown in Figures 3A,B, we confirmed the infection of target cells (Jurkat) by PCR for the Tax gene and the upregulation of CD25 expression, respectively. Surprisingly, 48 h after the target cell infection, a significant reduction in the percentage of PrP^C+^ cells (approximately 30%) was detected. This decrease was also detected at 96 h post-infection (Figure 3C), indicating that the infection may be directly related to the decrease in PrP^C^ expression in HTLV-1-infected cells. Additionally, we compared the Tax expression in MT-2 PrP^Cneg^ cells with MT-2 PrP^C+^ cells (sorted by FACS) by qRT-PCR. The results indicate that MT-2 PrP^Cneg^ cells expressed higher levels of Tax than MT-2 PrP^C+^ cells, reinforcing the relation between HTLV-1 infection and PrP^C^ modulation (Figure 3D).

### 3.4. Expression of the *Orf I* encoded HTLV-1 p12 protein is associated with decreased PrP^C^ levels

**Figure 4:**
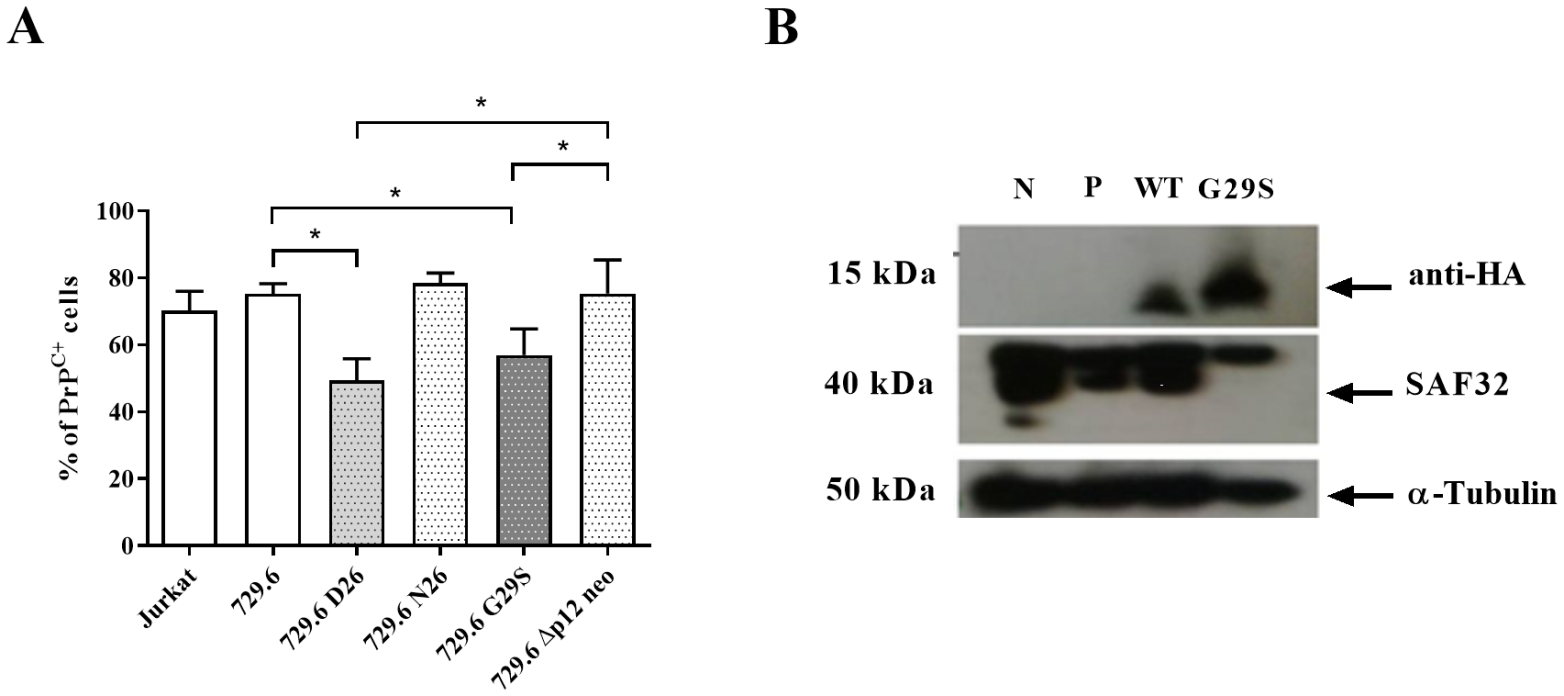
Role of p12 viral protein in PrP^C^ reduction. 5×10^5^ cells of Jurkat cells, B cell line 729.6, 729.6 D26 cells stably infected with pAB wild-type virus, 729.6 N26 (mutant that predominantly expresses the p8 viral protein), 729.6 G29S cells (predominantly expressing the p12 protein), and the 729.6 Δp12 cells (not expressing p8 or p12) were stained with the antibody SAF32 as described in the methods section. **(A)** PrP^C^ levels were analyzed by flow cytometry in Jurkat cells and 729.6 cells (non-infected cells) and transfected cells to compare the percentage of PrP^C+^. Statistical analysis was performed using the ANOVA statistical test following Bonferroni’s post-test. The means were considered significantly different when *p < 0.05. **(B)** 30 μg of total proteins from 10^6^ Jurkat cells or Jurkat cells transfected with cDNA plasmids from the *Orf I* region, wild-type plasmid (WT *Orf I* sequence), or G29S plasmid (mutant sequence of *Orf I*, which predominantly induces p12 production), cells without transfection (negative, N) and cells transfected with Pmax-GFP plasmid (P) were used as a control. Cells were separated in SDS-PAGE and transferred to nitrocellulose membrane to analyze PrP^C^ content by Western blot, using α-tubulin as a constitutive protein as described above.

The HTLV-1 viral genome encodes five orfs, among which *Orf I* encodes for the p12 protein that can subsequently be processed into p8 (Koralnik et al., 1992). The p12 and p8 proteins are involved in several functions in the infected cell, including the transmission of the virus to target cells and the regulation of host cell proteins (Albrecht et al., 2000; Nicot et al., 2004; Prooyen et al., 2010; Valeri et al., 2010; Sarkis et al., 2019). Because PrP^C^ is associated with cell activation and inflammation, we investigated the role of p12 and p8 in PrP^C^ expression. To achieve this goal, we used stably infected, virus-producing B cell line 729.6 (pAB wild-type virus, namely; 729.6 D26) and *Orf I* mutants: 729.6 N26 cells were characterized as a mutant that predominantly expresses the p8 protein; 729.6 G29S cells express predominantly p12 protein; and 729.6 Δp12 cells do not express p8 or p12 (Valeri et al., 2010). Like HTLV-1-infected Jurkat cells, 729.6 D26 showed a decreased percentage of PrP^C+^ cells compared to control cells (Figure 4A). This effect was observed in the p12-expressing 729.6 G29S cells, where a reduction of approximately 24% PrP^C+^ was detected. However, no significant reduction in PrP^C+^ cells was observed in 729.6 N26 cells or 729.6 Δp12 cells compared with the control parental 729.6 cells or Jurkat cells (Figure 4A).

To confirm that p12 was important for PrP^C^ reduction, Jurkat cells were transfected with *Orf I* expression plasmids, wild-type plasmid (WT *Orf I* sequence), and G29S plasmid (mutant sequence of *Orf I*, which predominantly induces p12 production) (Fukumoto et al., 2009). The levels of PrP^C^ were measured in Jurkat cells transfected with the WT or G29S plasmids. We detected a reduction in the PrP^C^ protein levels in WT and G29S-expressing Jurkat cells compared with controls (Figure 4B). Together, these results suggested that the p12 protein may play a relevant role in the downregulation of PrP^C^ in HTLV-1-infected cells.

### 3.5. Silencing of PrP^C^ does not affect HTLV-1 expression

**Figure 5:**
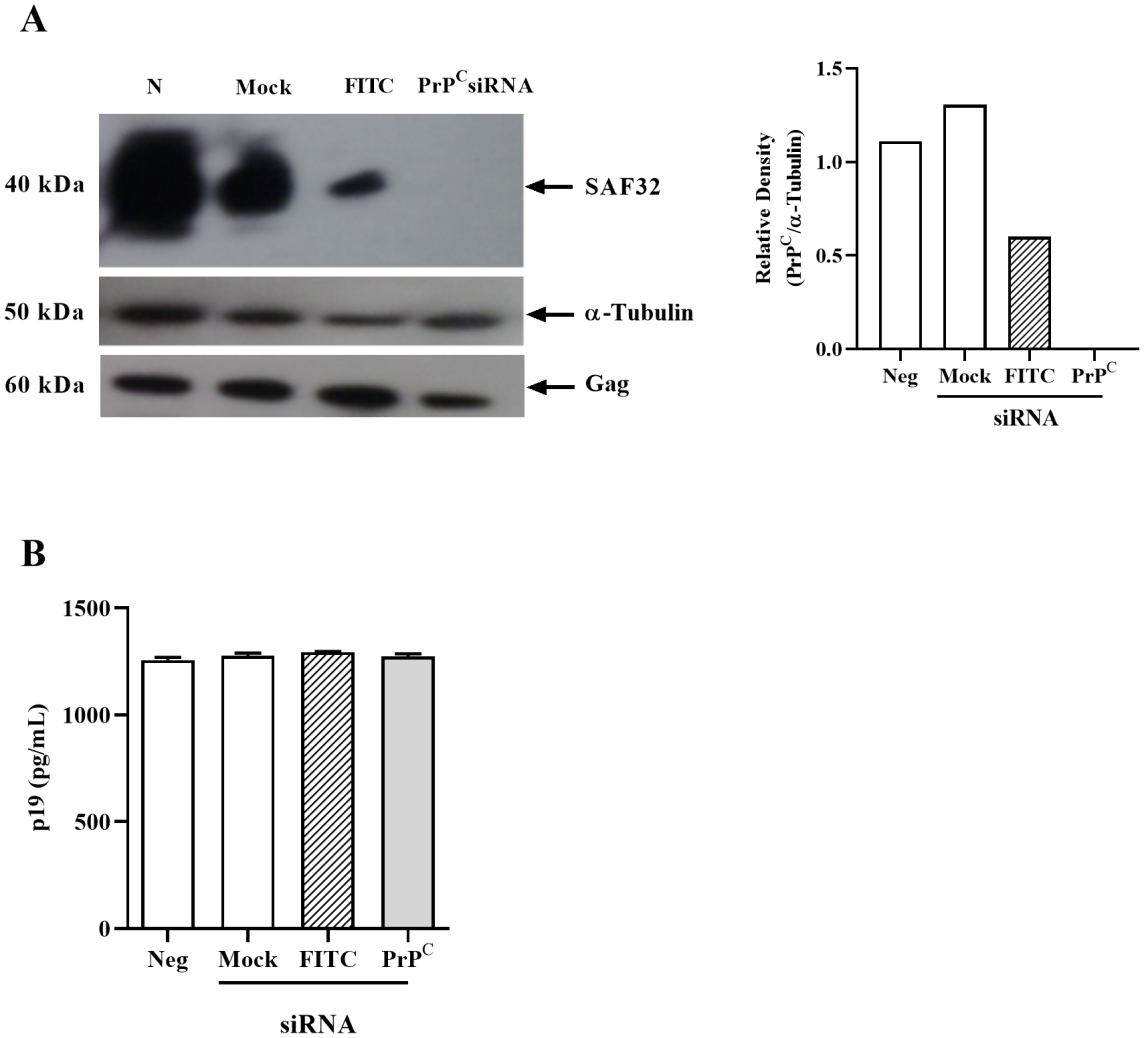
Effect of PrP^C^ silencing in HTLV-1-infected cells. **(A)** 10^6^ MT-2 cells were transfected with siRNA for PrP^C^ using lipofectamine 2000 and incubated for 24 h in RPMI medium at 37°C in a humid atmosphere with 5% CO_2_. Next, the cells were lysed with RIPA buffer, then 40 μg of total proteins were run on SDS-PAGE and nitrocellulose membrane transfer. The membrane was labeled with SAF32 and anti-α-tubulin antibodies. Relative densitometry of SAF32 in relation to α-tubulin was performed using ImageJ software. **(B)** p19 concentration in the supernatant of PrP^C^-silenced MT-2 cells determined by ELISA. Mean ± SEM from two independent experiments was represented in the graphic.

Finally, we investigated if reduced PrP^C^ expression affects production of the viral structural protein Gag. We used human PrP^C^ siRNA to target PrP^C^ expression in MT-2 cells. The siRNA to PrP^C^ significantly reduced PrP^C^ (SAF32) protein and mRNA expression compared with the FITC control or mock-transfected cells (Figure 5A). However, PrP^C^ silencing did not affect virus expression measured as p19 Gag in the supernatant (Figure 5B).

## 4. Discussion

PrP^C^ is involved in the pathogenesis of neurodegenerative diseases, such as dementia with Lewy bodies and Pick’s disease, inducing aggregates with α-synuclein, amyloid β aggregates, and tau protein (Corbett et al., 2020). Furthermore, increased levels of soluble PrP^C^ have been detected in the cerebrospinal fluid obtained from HIV-1-infected individuals with symptoms of cognitive disorders. This effect was also observed in the cerebrospinal fluid of monkeys infected with SIV, an animal model for the comparative study of infection with HIV-1, which correlated with the worsening of the severity of encephalopathy (Roberts et al., 2010). The biology of PrP^C^ was modulated by inflammatory cytokines and chemokines, such as IL-6, TNF-α, IL-8, and CCL4/MIP-1β, among others (Stoeck et al., 2014), which are also observed in patients with HAM/TSP (Champs et al., 2019; Souza et al., 2021; Freitas et al., 2022).

Our study has pioneered the investigation of PrP^C^ expression in CD4^+^ T lymphocytes from people living with HTLV-1. Our results demonstrate that infected individuals, both asymptomatic and HAM/TSP patients, have a reduced percentage of PrP^C+^ CD4^+^ T cells, representing a reduction between 20 and 25% compared with non-infected individuals. Corroborating these findings, Souza et al. (2021) demonstrated that patients with HAM/TSP have increased free PrP^C^ in the cerebrospinal fluid compared with asymptomatic patients, suggesting a shedding of PrP^C^ symptomatic individuals. The PrP^C^ shedding occurs during lymphocyte activation, cell–cell contact, and apoptosis (Zhang et al., 2020). In addition, CCL2 and TNF-α (200 ng/mL and 10 ng/mL) stimulation induces PrP^C^ shedding in astrocytes *in vitro* (Megra et al., 2017). The authors connected PrP^C^ shedding with ADAM10 metalloprotease activation induced by these cytokines (Megra et al., 2017). In PBMCs stimulated with a low dose of TNF-α (1 ng/mL), we did not observe a reduction in the percentage of PrP^C^-positive cells (data not shown).

Using HTLV-1-infected cells (MT-2, C91-PL, and ED40515[−]), we observed a decrease in the percentage of PrP^C+^ cells and protein levels. The results were confirmed by *in vitro* infection of Jurkat and 729.6 cells, which also promote a reduction in PrP^C^ levels. No differences were found in the *prnp* gene transcript levels in CD4^+^ T cells obtained from infected and uninfected individuals, or in cell lines, suggesting that PrP^C^ expression is regulated in infected cells by a post-transcriptional event. Thus, we reasoned that the downmodulation of PrP^C^ could be a result of the activity of viral proteins such as p12 and p8. Using cells transfected with different constructs, we demonstrated that the p12 viral protein is related to the downregulation of PrP^C^. In agreement with our findings, it has already been described that p12 induces a decrease in the expression of other molecules in cells infected by HTLV-1; p12 is capable of binding to the heavy chain of the MHC-I molecule in the rough endoplasmic reticulum and preventing association with β-2 microglobulin (mβ-2). The absence of binding of the MHC-I heavy chain with mβ-2 induces the translocation of p12-associated MHC-I to the proteasome in the cytosol, promoting the degradation of the complex. Consequently, the infected cell presents a reduction in the expression of MHC-I on the cell surface and reduces the presentation of HTLV-1 antigens, making it less susceptible to the action of cytotoxic cells (Johnson et al., 2001; Pise-Masison et al., 2014). Using a reversible inhibitor of the proteasome, MG-132 (5 mM), in transfected Jurkat cells with WT p12 plasmid (D26) we did not observe any modification in PrPC levels (Supplementary Figure 4). In addition, the p12 viral protein alters the distribution and expression of LFA-1 and ICAM-1. The expression of these adhesion molecules occurs in cholesterol-rich regions of the plasma membrane (lipid raft domain), as does the PrP^C^ (Kim et al., 2006; Banerjee et al., 2007; Westergard et al., 2007; Prooyen et al., 2010).

It was previously reported that PrP^C^ expression in HEK293 cells reduces the expression of HIV Pr55Gag and viral particle production (Leblanc et al., 2004). The anti-HIV properties of PrP^C^ were linked to its binding to the viral genome and reducing translation (Alais et al., 2012). Thus, the reduction of PrP^C^ favors HIV replication. Moreover, PrP^C^ dysregulation was detected in cognitively impaired HIV-1-infected individuals, suggesting its contribution to the pathogenesis of HIV-1-associated CNS disease. Indeed, increased levels of soluble PrP^C^ were observed in the cerebrospinal fluid of patients with HIV-associated neurocognitive impairment. In addition, after *in vitro* PrP^C^ addition to cultures, an increase of both CCL2 and IL-6 production by astrocytes was reported, suggesting that PrP^C^ is a biomarker of HIV-associated neurocognitive impairment (Roberts et al., 2010).

In contrast, the reduction of PrP^C^ in HTLV-1-infected cells was not associated with decreased viral production. PrP^C^ silencing neither altered the production of HTLV-1 viral particles in MT-2 cells nor significantly impacted viral transmission to Jurkat cells. It is well known that HTLV-1 infection induces the activation of T lymphocytes, leading to spontaneous proliferation, expression of molecules associated with cell activation, and the production of pro-inflammatory cytokines (Prince et al., 1994; Novaes et al., 2013; Coutinho et al., 2014; Futsch et al., 2018). Studies using the murine experimental encephalomyelitis (EAE) model have shown that PrP^C^-deficient animals (knockout or silenced) show increased transcripts and secretion of pro-inflammatory cytokines such as IL-17 and IFN-γ, as well as a significant enhancement in the expression of transcription factors Tbet and RORγt (Tsutsui et al., 2008; Hu et al., 2010). Consistent with those findings, we found an increase in the percentage of IFN-γ^+^ cells in the PrP^C^-negative CD4^+^ cell population obtained from people living with HTLV-1.

In conclusion, we have shown that HTLV-1 infection induces a reduction in PrP^C^ levels, linked to viral protein p12. Moreover, a reduction of PrP^C^ was also observed in lymphocytes from people living with HTLV-1, significantly higher in IFN-γ-producing cells. These findings may be linked to increased levels of PrP^C^ in CSF, suggesting that it could be included as a biomarker for HAM/TSP.

## Data availability statement

The original contributions presented in the study are included in the article/Supplementary material. Further inquiries can be directed to the corresponding author.

## Ethics statement

The studies involving human participants were reviewed and approved by the Comitê de Ética em Pesquisa do Instituto Nacional de Infectologia Evandro Chagas – INI/FIOCRUZ. The patients/participants provided their written informed consent to participate in this study.

## Author contributions

RL, IS, GF, CP-M, and JE-L designed the experiments, analyzed the data, and wrote the manuscript with the collaboration of all authors. IS, AG, and RM carried out experiments and analyzed the data. OE carried out assays of HTLV-1 proviral load and obtained ethics approval. ML and AL carried out clinical evaluation and supplied blood samples. All authors reviewed and approved the manuscript.

## Funding

This study was supported by grants from Fundação Carlos Chagas Filho de Amparo à Pesquisa do Estado do Rio de Janeiro (FAPERJ), Fundação Oswaldo Cruz, and the Brazilian Ministry of Health. AG and IS were supported by Postdoc Research Fellowships from Coordenação de Aperfeiçoamento de Pessoal de Nível Superior (CAPES). Part of this study was supported by the Intramural Research Program, Center for Cancer Research, National Cancer Institute, NIH, Bethesda, Maryland, United States.

## Conflict of interest

The authors declare that the research was conducted in the absence of any commercial or financial relationships that could be construed as a potential conflict of interest.

## Publisher’s note

All claims expressed in this article are solely those of the authors and do not necessarily represent those of their affiliated organizations, or those of the publisher, the editors and the reviewers. Any product that may be evaluated in this article, or claim that may be made by its manufacturer, is not guaranteed or endorsed by the publisher.
