# Supplementary material for: HTLV-1 p12 modulates the levels of prion protein (PrPC) in CD4+ T cells
Source: Front Microbiol. 2023 Aug 10;14:1175679. doi: 10.3389/fmicb.2023.1175679 (PMC10449582; doi:10.3389/fmicb.2023.1175679)
Supplement: Supplementary file 1 [file Data_Sheet_1.docx]

**Supplemental Methods**

Jurkat cells (3×10^6^) were transfected with 2 μg of DNA plasmid using Amaxa® Cell Line Nucleofector® Kit V. The HTLV-1 molecular clones p12(D26) and p8(N26) and pME-WT were described previously (24,30,32,44). Electroporation was performed by Nucleofector II Amaxa equipment (Lonza, Switzerland) according to manufacturer’s instructions. The transfected cells were incubated with or without a reversible inhibitor of the proteasome, MG-132 (5 µM) for 6h at 37°C in a humid atmosphere with 5% of CO_2_. Following, cells were washed with PBS, centrifuging for 7 minutes at 200 g at 4°C. Cells were incubated with LIVE/DEAD Fixable staining (1:1000, 405 nm) in PBS for 30 minutes and washed with PBS containing 1% SFB and 0.01% sodium azide. Next, cells were incubated with the PrP^C^ Monoclonal Antibody (Clone SAF 32, 1:50) and biotinylated for 30 minutes at 4°C. cells were washed with PBS + 5% FBS, centrifuging for 7 minutes at 200 g at 4°C. Cells were then stained with streptavidin conjugated to PE for 30 minutes at 4°C. Cells were washed and fixed with 2% paraformaldehyde, washed with PBS, and permeabilized with a permeabilization solution (eBioscience/Thermo Fisher Scientific) for 30 minutes at room temperature. Next, the cells were labeled with the antibodies for 30 minutes at room temperature. Following this, cells were washed, and 30,000 events were acquired for analysis of the percentage and mean fluorescence intensity, using FORTESSA (BD) cytometer. Flow Jo V10 software was used for analysis.

**Supplemental Legends**

**Supplemental Figure 1.** **PrP^C^ levels in cell lines.** 5x10^5^ cells of different cell lines were stained with the antibody SAF32 as described in the methods section. PrP^C^ levels were analyzed by flow cytometry in Jurkat cells (noninfected cells) and MT-2 cells (HTLV-1-infected cells) to compare the percentage of PrP^C+^. The percentage in the histograms corresponds to PrP^C^-stained cells (blue to Jurkat and orange to MT-2 cells). Black and gray histograms represent the negative control and secondary control cells, respectively.

**Supplemental Figure 2. Percentage of CD4^+^PrP^C+^ cells from people living with HTLV-1 infected individuals.** 5x10^5^ cells of PBMCs obtained from HTLV-1 carrier were stained with the antibody SAF32, lineage-specific antibodies (anti-CD4, -CD8), anti-human IFN-γ, and anti-human IL-17 as described in the methods section. Gating strategy for IFN-γ^+^ or IL-17^+^ in PrPC^neg^ CD4^+^ cells or PrPC^+^ CD4^+^ cells analysis.

**Supplemental Figure 3. Percentage of CD4^+^PrP^C+^ cytokine producers’ cells from people living with HTLV-1.** 5x10^5^ cells of PBMCs obtained from HTLV-1 carriers (n=6) were stained with the antibody SAF32 and lineage-specific antibodies as described in the methods section. (**A**) Percentage of IL-4^+^ in PrP^Cneg^ CD4^+^ cells or PrP^C+^ CD4^+^ cells from PBMCs of people living with HTLV-1. (**B**) Percentage of IL-6^+^ in PrP^Cneg^ CD4^+^ cells or PrP^C+^ CD4^+^ cells from PBMCs of people living with HTLV-1. (**C**) Percentage of IL-10^+^ in PrP^Cneg^ CD4^+^ cells or PrP^C+^ CD4^+^ cells from PBMCs of people living with HTLV-1. The significance was determined by Mann-Whitney test. Each symbol represents one donor, and the bar indicates the median value.

## Supplemental Figure 4. Proteasome inhibition did not alter PrP^C^ levels. 5x10^5^ cells of Jurkat cells were transfected with D26 (wild-type virus, p12WT), N26 (mutant that predominantly expresses the p8 viral protein), pME (control), and treated with a reversible inhibitor of the proteasome, MG-132 (5 µM) for 6 h. Following this, noninfected cells and transfected cells were stained with the antibody SAF32 as described in the methods section, and PrP^C^ levels were analyzed by flow cytometry.
