# Supplementary figures and images for: HTLV-1 p12 modulates the levels of prion protein (PrPC) in CD4+ T cells
Source: Front Microbiol. 2023 Aug 10;14:1175679. doi: 10.3389/fmicb.2023.1175679 (PMC10449582; doi:10.3389/fmicb.2023.1175679)

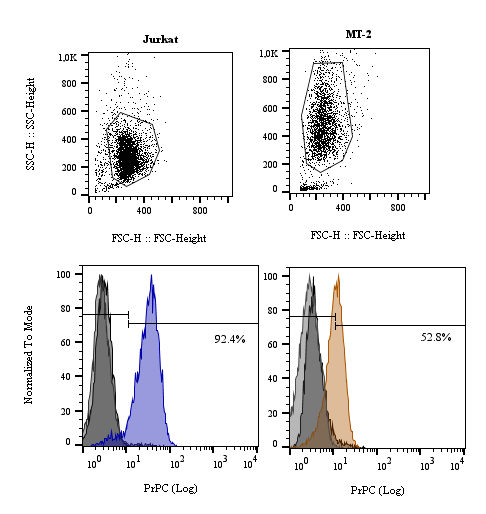

Supplement: Supplementary file 2 [file Image_1.TIFF]

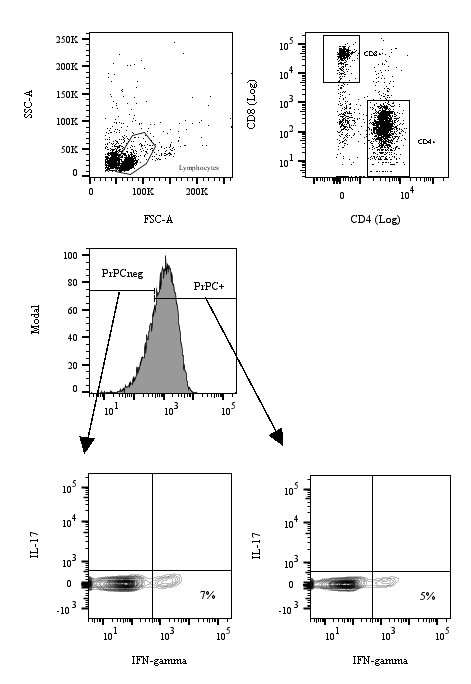

Supplement: Supplementary file 3 [file Image_2.TIFF]

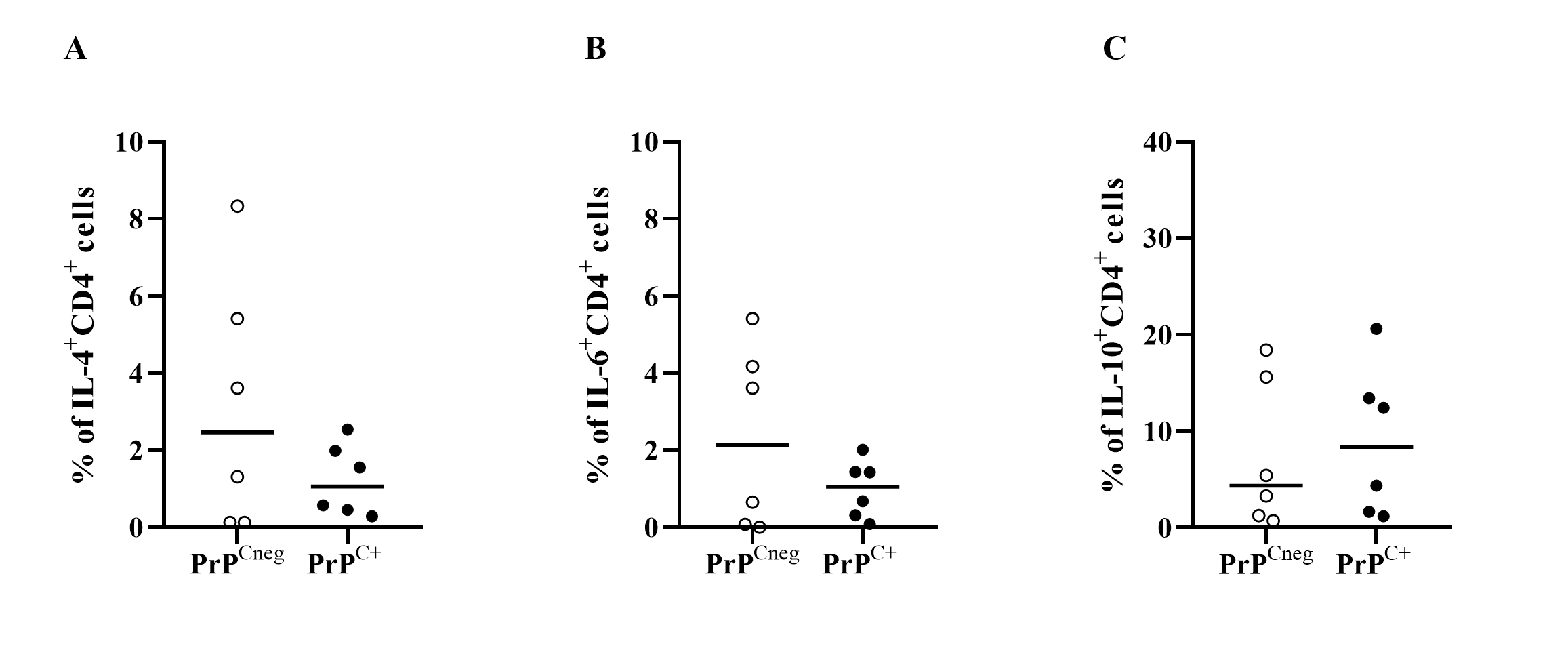

Supplement: Supplementary file 4 [file Image_3.tif]

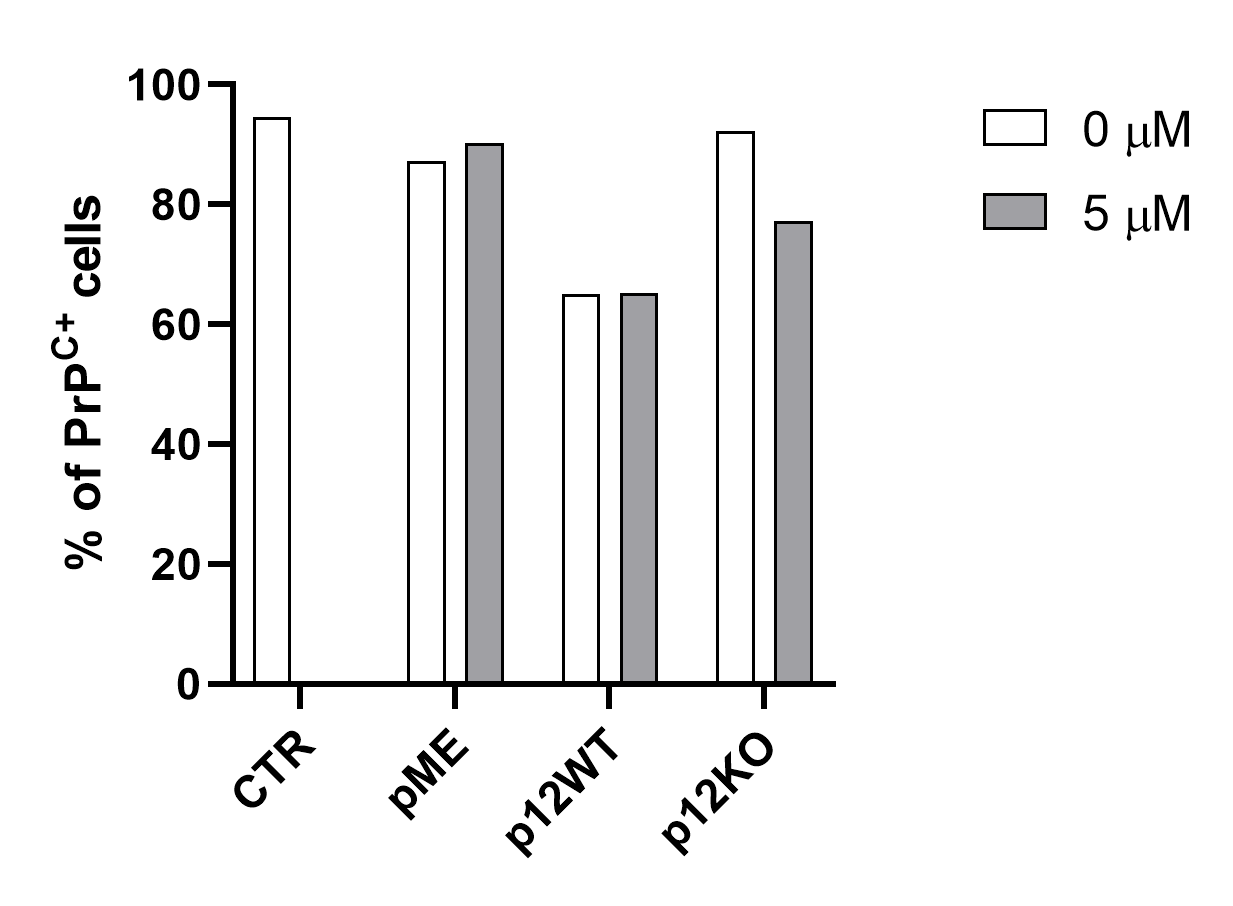

Supplement: Supplementary file 5 [file Image_4.tif]
